# Supplementary material for: Identification of antimicrobial peptides from ancient gut microbiomes
Source: Nat Commun. 2026 Jan 14;17:1788. doi: 10.1038/s41467-026-68495-0 (PMC12917264; doi:10.1038/s41467-026-68495-0)
Supplement: Supplementary file 1 — Supplementary Information [file 41467_2026_68495_MOESM1_ESM.pdf]

Supplementary Information

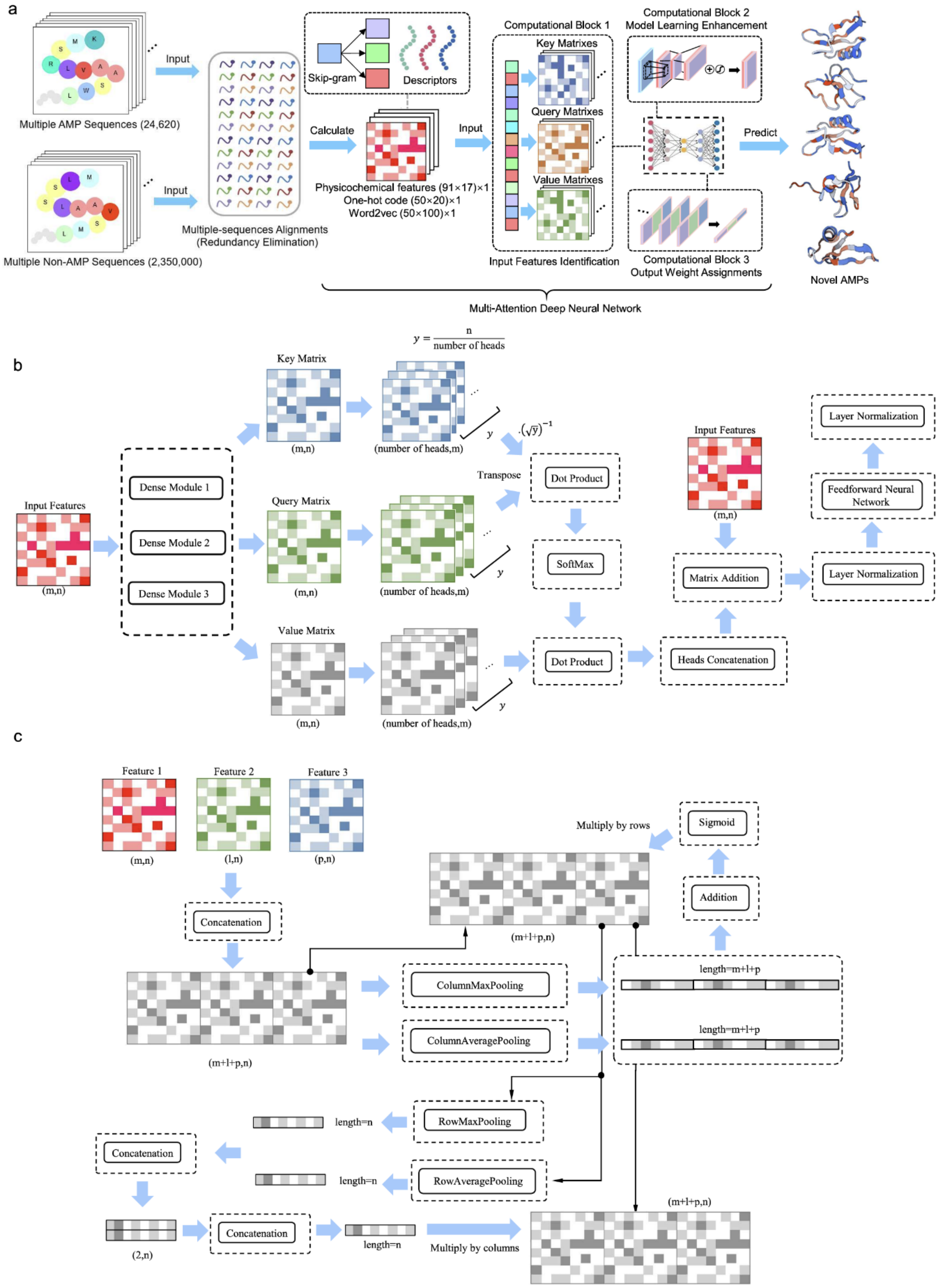

Figure S1. a. The overall computational framework of the multi-attention deep neural network AMPLiT, in which three computational blocks were embedded in core structures for improving the predicting capabilities and accelerating the fitting processes. **b.** The framework of the computational block 1. The computational

block 1 was architected following the fundamental paradigm of multi-head attention mechanisms. This framework was embedded in our model for achieving dual objectives of predictive efficiency and balanced computational costs. **c.** The framework of the computational block 3. The computational block 3, a novel computational module, was mathematically designed in the framework; It dynamically refines informative features while suppressing less useful noises. This computational block was embedded in our model for achieving dual objectives of reasonable predictive efficiency and balanced computational costs.

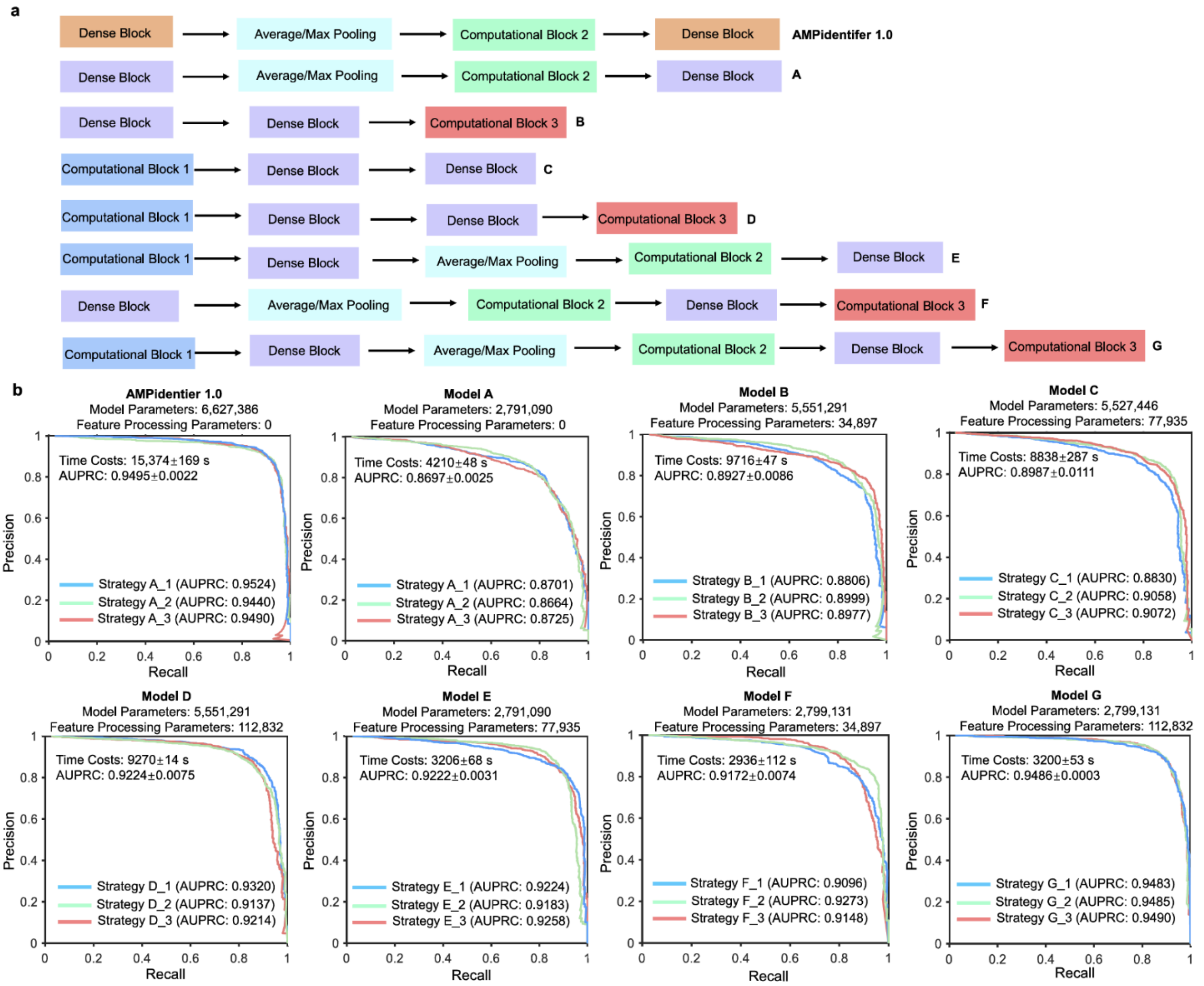

Figure S2. The comparisons of AMPLiT performances by using different modules and frameworks. a. The summary of the model frameworks tested in this study. **b.** The performances of different model frameworks in comparison with AMPidentifier 1.0 by using indicators of AUPRC, scales of model parameters, and training time costs, with three replicates in each group. All tests were conducted on consumer-grade portable devices (Intel i7-10875H CPU).

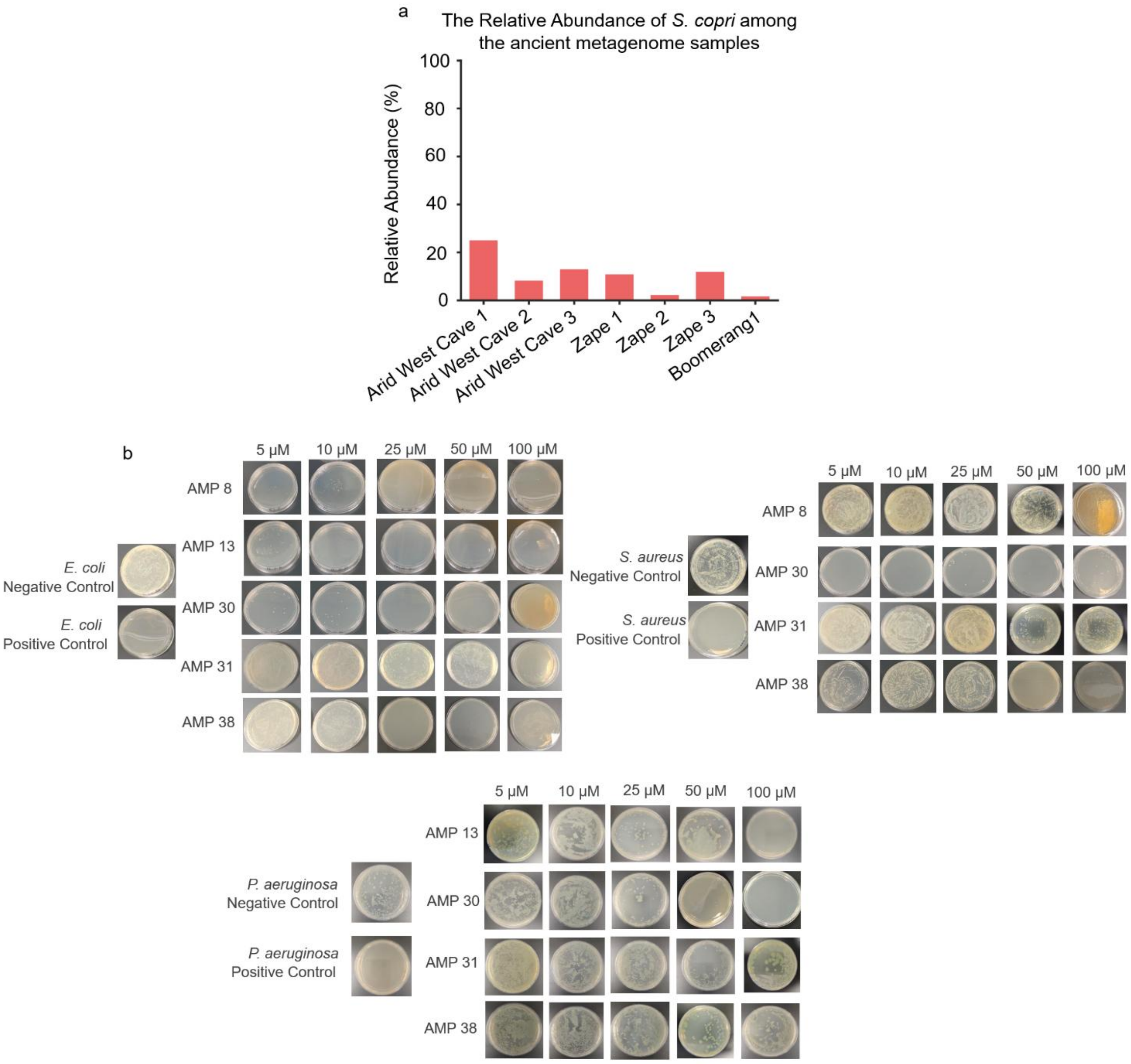

Figure S3. a. The abundances of *S. copri* among the 7 ancient stool metagenomic samples. The statistical results revealed high prevalences of *S. copri* in ancient *Homo sapiens*. Results were calculated by MetaPhlan 4.0 [1]. **b.** The representative plate spreading images of antimicrobial efficacy tests for lead AMPs identified from *S. copri*.

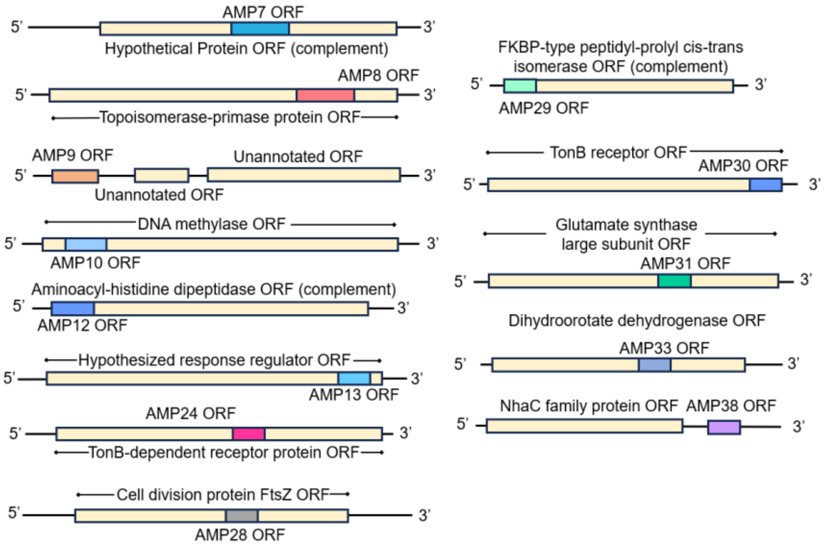

Figure S4. The annotations of the assembled ancient metagenomic contigs that harbors representative AMP sequences. The molecular origins of representative AMPs in ancient metagenomic contigs that have been phylogenetically traced to ancient human gut commensal *S. copri*.

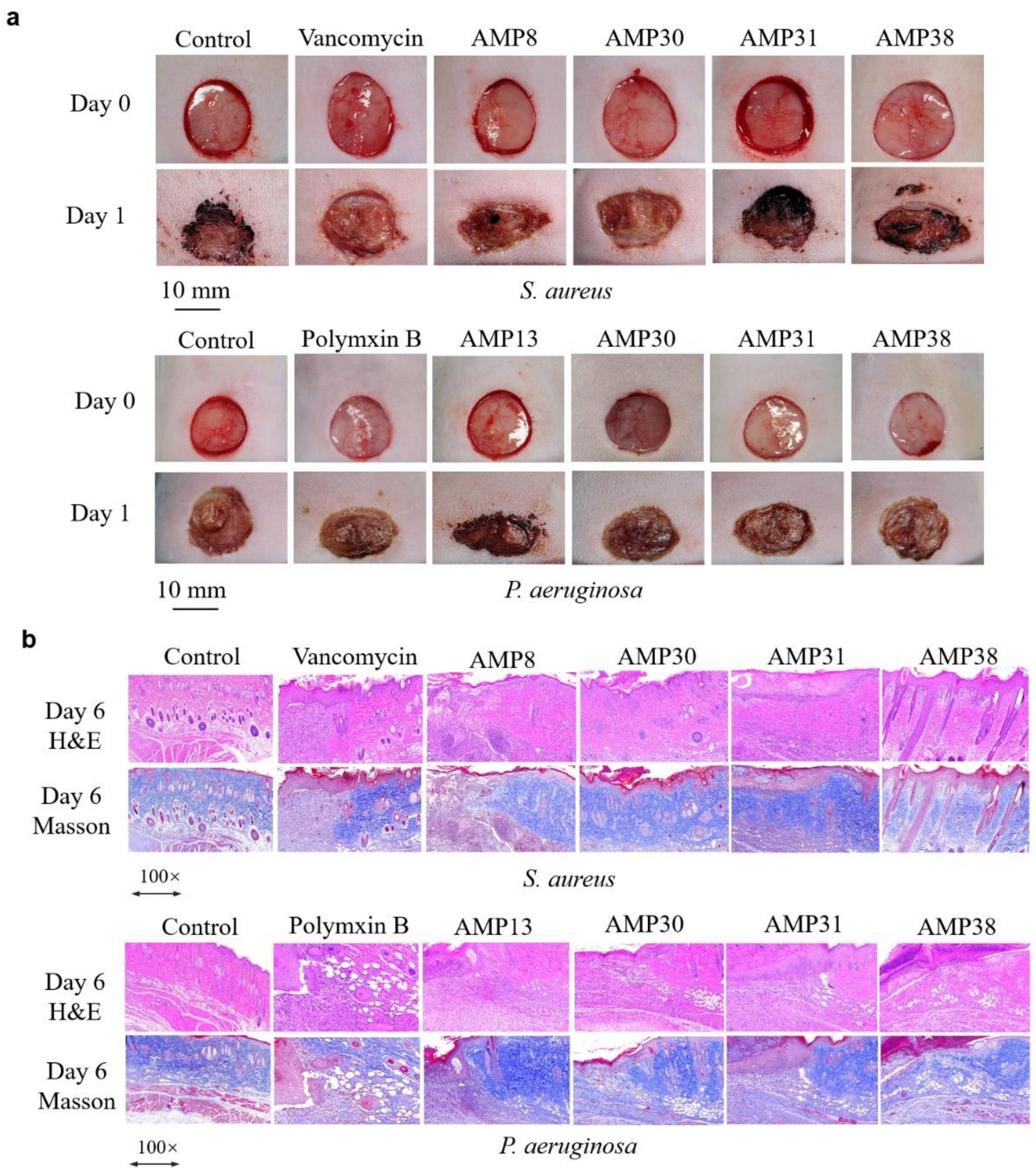

Figure S5. The supplementary data of antimicrobial test. a. Representative *in vivo* wound healing results by negative control PBS, AMP groups, and positive control vancomycin or polymyxin B at the same dosage *in vivo*. **b.** The representative histological results of mice wound tissues from groups of control, AMP8, AMP13, AMP30, AMP31, AMP38, and vancomycin/polymxin B groups, generated using H&E and Masson staining protocols.

Table S1. The AI-predicted 40 peptides chemically synthesized in this study

AMP Number	Sequence	AI score	Length
1	KERKKYGLKAARRAPQFSKR	0.999996	20
2	ASTQEVLVVPSQFFKR	0.999567	16
3	NSARKSSKTSSSAISQSSW	1	19
4	FGGVRIQICGEATREDIRP	0.999981	19
5	HHPSPQSSLPTATGRRGPRC	0.962317	20
6	WVNRTSVRSRSRWLPTRD	0.999918	18
7	TTLPRNITSRRSCRP	0.998244	15
8	AIRSLTIRIYTLATRT	0.999972	16
9	KSTKSRLASLFNSR	0.999996	14
10	KATNPPSLTVSRALTKK	0.999974	17
11	LSFRRCFLLYAI	0.995119	12
12	VWDHLLEILRSVAK	0.999442	14
13	KRVKIERWLLCQR	0.999999	13
14	VPRPRTRSRPLSAPG	0.999999	15
15	KCSLLPLHSISAR	1	13
16	SGKTGRNSCKFCSRIAF	0.97907	17
17	NRLSRSRTDWLPTLR	0.993535	15
18	PIRLPNANGNWQKRRKS	0.999589	17
19	VQPIGQYRSSNRRLW	0.950045	15
20	WLVSWPTRPVLREWYR	1	16
21	KSCKAHSTSRYCRNNHT	0.999938	17
22	VICPANPKHKQRQG	0.999879	14
23	HQRHLTSRKLDLT	0.999934	13
24	KLRVWYSRLRYSP	0.999975	13
25	DLLVLSKVLFLSW	0.949187	13
26	AQQDFEHIKFHFTSEF	0.967584	16
27	TSISRHQKRPSRN	0.999999	13
28	HPLRLSPSRHKHRKDVRC	1	18
29	VLKSYKRASGKKWITE	0.950951	16
30	PLKYPSSYYSRWRGQVGLKYTF	0.999843	22
31	PASVCRLPSSLRVVRFVR	1	18
32	LTSWNCWKILASKKKLLR	1	18
33	PYRHLSRLDKRYSAR	0.998293	15
34	VISATKPEKPGKPRLAKPATA	0.99348	21
35	LSQSTSILANFALKDILR	0.924391	18
36	SGHRHCSQPSTKASSRFSPRSLPRW	0.999999	25
37	SERFSLKNSSEPATRPPRKA	1	20
38	PKLSARSLVKRLR	0.999995	13
39	FLRIVTIQIIIFVFG	0.996848	15
40	LEDKDENKYREAF	0.952109	13

Table S2. MIC of representative lead peptides measured by CFU plate spreading method (μM)

AMP	*E. coli 25404*	*S. aureus 6538*	*P. aeruginosa 27853*
AMP 8	25	100	-
AMP 13	10	-	100
AMP30	25	5	100
AMP31	100	>100	>100
AMP38	25	50	>100

Supplementary References:

1. Blanco-Míguez, A., *et al.* Extending and improving metagenomic taxonomic profiling with uncharacterized species using MetaPhlAn 4. *Nature Biotechnology* **41**, 1633-1644 (2023).
2. Chen, S., *et al.* Screening and identification of antimicrobial peptides from the gut microbiome of cockroach Blattella germanica. *Microbiome* **12**, 272 (2024).
