# Supplementary material for: Identification of antimicrobial peptides from ancient gut microbiomes
Source: Nat Commun. 2026 Jan 14;17:1788. doi: 10.1038/s41467-026-68495-0 (PMC12917264; doi:10.1038/s41467-026-68495-0)
Supplement: Supplementary file 2 — Reporting Summary [file 41467_2026_68495_MOESM2_ESM.pdf]

nature portfolio

Corresponding author(s): Qi Su

Last updated by author(s): Dec 18, 2025

Reporting Summary

Nature Portfolio wishes to improve the reproducibility of the work that we publish. This form provides structure for consistency and transparency in reporting. For further information on Nature Portfolio policies, see our Editorial Policies and the Editorial Policy Checklist.

Statistics

For all statistical analyses, confirm that the following items are present in the figure legend, table legend, main text, or Methods section.

n/a	Confirmed	
☐	☒	The exact sample size (*n*) for each experimental group/condition, given as a discrete number and unit of measurement
☐	☒	A statement on whether measurements were taken from distinct samples or whether the same sample was measured repeatedly
☐	☒	The statistical test(s) used AND whether they are one- or two-sided<br>*Only common tests should be described solely by name; describe more complex techniques in the Methods section.*
☐	☒	A description of all covariates tested
☐	☒	A description of any assumptions or corrections, such as tests of normality and adjustment for multiple comparisons
☐	☒	A full description of the statistical parameters including central tendency (e.g. means) or other basic estimates (e.g. regression coefficient) AND variation (e.g. standard deviation) or associated estimates of uncertainty (e.g. confidence intervals)
☐	☒	For null hypothesis testing, the test statistic (e.g. *F*, *t*, *r*) with confidence intervals, effect sizes, degrees of freedom and *P* value noted<br>*Give P values as exact values whenever suitable.*
☒	☐	For Bayesian analysis, information on the choice of priors and Markov chain Monte Carlo settings
☐	☒	For hierarchical and complex designs, identification of the appropriate level for tests and full reporting of outcomes
☐	☒	Estimates of effect sizes (e.g. Cohen's *d*, Pearson's *r*), indicating how they were calculated

Our web collection on statistics for biologists contains articles on many of the points above.

Software and code

Policy information about availability of computer code

Data collection	The original codes of AMPLiT were publicly available at Github: https://github.com/ChenSizhe13893461199/AMPLiT. All involved sequence data were collected from publicly available datasets (UniProt, CAMP, LAMP, APD3, and etc.). The utilization of previously published metagenomic samples were all clearly stated in the manuscript. All these information have been clearly stated in the current manuscript.
Data analysis	All data analysis were conducted by using Python 3.9 and MATLAB R2024a, with clear descriptions provided in the current Manuscript.

For manuscripts utilizing custom algorithms or software that are central to the research but not yet described in published literature, software must be made available to editors and reviewers. We strongly encourage code deposition in a community repository (e.g. GitHub). See the Nature Portfolio guidelines for submitting code & software for further information.

Data

Policy information about availability of data

All manuscripts must include a data availability statement. This statement should provide the following information, where applicable:

- Accession codes, unique identifiers, or web links for publicly available datasets
- A description of any restrictions on data availability
- For clinical datasets or third party data, please ensure that the statement adheres to our policy

All data and methods relevant to this research are provided in the Supplementary Information. Descriptions of further supplementary information are also provided in the Supplementary Information. The original codes of AMPLiT were publicly available at Github: https://github.com/ChenSizhe13893461199/AMPLiT. No clinical datasets were used in this study.

Research involving human participants, their data, or biological material

Policy information about studies with human participants or human data. See also policy information about sex, gender (identity/presentation), and sexual orientation and race, ethnicity and racism.

Reporting on sex and gender	No information relevant to sex and gender are used in this study
Reporting on race, ethnicity, or other socially relevant groupings	No information relevant to race, ethnicity, or other socially relevant groupings are used in this study.
Population characteristics	No information relevant to population characteristics are used in this study.
Recruitment	No information relevant to recruitment are used in this study.
Ethics oversight	Not applicable to the study, as this study didn't involve human participants.

Note that full information on the approval of the study protocol must also be provided in the manuscript.

Field-specific reporting

Please select the one below that is the best fit for your research. If you are not sure, read the appropriate sections before making your selection.

- [x] Life sciences
- [ ] Behavioural & social sciences
- [ ] Ecological, evolutionary & environmental sciences

For a reference copy of the document with all sections, see nature.com/documents/nr-reporting-summary-flat.pdf

Life sciences study design

All studies must disclose on these points even when the disclosure is negative.

Sample size	7 previously published ancient metagenomic samples, and 3 environmental samples were used in this study.
Data exclusions	Those metagenomic samples stated with "severe pollution" were excluded, according to previously published article "Reconstruction of ancient microbial genomes from the human gut. Nature 594, 234-239 (2021)"
Replication	For both in vitro and in vivo experiments, all tests were replicated for at least 3 times and all tests revealed successful robustness.
Randomization	For animal tests, all animals were randomly divided into different treatment groups.
Blinding	The "blinding" procedures to data allocation were not necessary for this study.

Reporting for specific materials, systems and methods

We require information from authors about some types of materials, experimental systems and methods used in many studies. Here, indicate whether each material, system or method listed is relevant to your study. If you are not sure if a list item applies to your research, read the appropriate section before selecting a response.

Materials & experimental systems

n/a	Involved in the study	
x		Antibodies
	x	Eukaryotic cell lines
x		Palaeontology and archaeology
	x	Animals and other organisms
x		Clinical data
x		Dual use research of concern
x		Plants

Methods

n/a	Involved in the study	
x		ChIP-seq
x		Flow cytometry
x		MRI-based neuroimaging

Eukaryotic cell lines

Policy information about cell lines and Sex and Gender in Research

Cell line source(s)	Caco-2 intestine model cells were commercially purchased from CELLCOOK
Authentication	The involved cell lines have been authenticated by commercial supplier and no further auuthentication was conducted in the laboratory.

Mycoplasma contamination	All cell lines were routinely tested negative for mycoplasma contamination before experiments.
Commonly misidentified lines (See ICLAC register)	No such lines were used in the study.

Animals and other research organisms

Policy information about studies involving animals; ARRIVE guidelines recommended for reporting animal research, and Sex and Gender in Research

Laboratory animals	For animal study, a total of 108 healthy female SD rats (6-8 weeks, 190-220 g, purchased from SPF(Beijing)biotechnology co.,Ltd.) were used in assessing efficacy of antimicrobial peptides, with 12/12 dark/light cycle, room temperature and humidity.
Wild animals	No wild animals have been used in the study.
Reporting on sex	The sex-based information and analysis is not relevant to the topic of the study.Only female rats were used in this study to minimize sex-related variability in wound healing and immune response.
Field-collected samples	The study didn't involve samples collected from filed.
Ethics oversight	All experiments using animals were consistent with the ethical policies and all involved experimental protocols were approved by the Laboratory Animal Welfare & Ethics Committee at the Health Science Center of Kunming Medical University (New Permit Number: 2025DF007).

Note that full information on the approval of the study protocol must also be provided in the manuscript.

Plants

Seed stocks	*Report on the source of all seed stocks or other plant material used. If applicable, state the seed stock centre and catalogue number. If plant specimens were collected from the field, describe the collection location, date and sampling procedures.*
Novel plant genotypes	*Describe the methods by which all novel plant genotypes were produced. This includes those generated by transgenic approaches, gene editing, chemical/radiation-based mutagenesis and hybridization. For transgenic lines, describe the transformation method, the number of independent lines analyzed and the generation upon which experiments were performed. For gene-edited lines, describe the editor used, the endogenous sequence targeted for editing, the targeting guide RNA sequence (if applicable) and how the editor was applied.*
Authentication	*Describe any authentication procedures for each seed stock used or novel genotype generated. Describe any experiments used to assess the effect of a mutation and, where applicable, how potential secondary effects (e.g. second site T-DNA insertions, mosiacism, off-target gene editing) were examined.*
